# Changes in Substance Use Diagnoses in the Great Plains during the COVID-19 Pandemic

**DOI:** 10.3390/healthcare12161630

**Published:** 2024-08-16

**Authors:** Ahmed Nahian, Lisa M. McFadden

**Affiliations:** 1 College of Osteopathic Medicine, Lake Erie College of Osteopathic Medicine at Seton Hill, Lynch Hall, 20 Seton Hill Dr, Greensburg, PA 15601, USA; 2 Division of Basic Biomedical Sciences, University of South Dakota, 414 E. Clarke St., Vermillion, SD 57069, USA

**Keywords:** electronic health records, great plains, opioid use disorder, alcohol use disorder, stimulant use disorder

## Abstract

As drug overdose mortality rises in the United States, healthcare visits present critical opportunities to mitigate this trend. This study examines changes in healthcare visits for substance use disorders (SUDs) and remission prior to and during the COVID-19 pandemic in the Great Plains, with a focus on identifying the characteristics of those served. Data were analyzed from 109,671 patient visits (mode = one visit per patient), encompassing diverse demographics, including sex, age, race, ethnicity, and geographic location. Visits analyzed included those for Alcohol Use Disorder (AUD), Opioid Use Disorder (OUD), or Stimulant Use Disorder (StUD) and those in remission of these disorders between March 2019 and March 2021. Patient demographic information and geographic factors, like rurality and Medicaid expansion status, were considered, and logistic regression was utilized. Visits were primarily by White (70.83%) and Native American (21.39%) patients, non-Hispanic (91.70%) patients, and males (54.16%). Various demographic, geographic, and temporal trends were observed. Findings indicated that males were more likely to receive an AUD diagnosis, while females were more likely to receive an OUD or StUD diagnosis. Metropolitan-residing patients were more likely to receive an AUD diagnosis, while non-metropolitan patients were more likely to receive an OUD diagnosis. Remission odds increased for StUD during the pandemic but decreased for AUD and OUD. These findings illuminate the demographic and geographic patterns of SUD-related healthcare visits, suggesting critical touchpoints for intervention. The results emphasize the urgent need for targeted healthcare strategies, especially in rural and underserved areas, to address persistent health disparities.

## 1. Introduction

The onset of the COVID-19 pandemic instigated unprecedented changes worldwide, necessitating various measures to curb the virus’s spread. These measures included the closure of schools and workplaces, limitations on in-person healthcare services, travel restrictions, and encouragement of limited social interactions [1,2]. While effective in reducing SARS-CoV-2 transmission, these interventions also led to significant consequences for mental and physical health. Research indicated that stay-at-home policies were associated with increased levels of depression, anxiety, and other mental health issues, particularly impacting individuals with pre-existing mental health disorders and the elderly [3,4,5,6]. Additionally, there was a notable rise in deaths from various non-COVID-19-related conditions, including cardiac arrests, liver disease, diabetes, and incidents like accidents, motor vehicle accidents, drug overdoses, assaults, and homicides, especially among younger adults [7,8,9,10]. Thus, while social distancing measures were essential for controlling the virus, they inadvertently affected overall well-being.

Substance use disorders (SUDs) have long been a critical public health issue [11], and the COVID-19 pandemic introduced additional challenges for those struggling with SUDs [12]. The pandemic exacerbated social isolation, financial strain, and generalized anxiety, further complicating the lives of individuals with SUDs [12,13]. Moreover, the healthcare system faced increased strain and disruption, hindering access to essential SUD treatment services [14,15]. It is crucial to understand how the pandemic affected individuals with SUDs to inform policies and practices that can improve care, particularly for diverse and underserved populations.

The Great Plains region of the United States, known for its vast agricultural areas and low population density, encounters significant challenges in providing accessible mental health and SUD treatments. These challenges are exacerbated by the region’s rural nature, which often results in limited healthcare infrastructure and services. Consequently, residents frequently experience barriers to timely and adequate care [16,17,18]. Initial studies during the pandemic highlighted a significant impact on healthcare in this region, with notable reductions in total healthcare visits and modest increases in telehealth usage [19]. Factors such as race, ethnicity, and geographical barriers further influence substance use outcomes in rural settings [20]. The region’s social determinants of health, including disparities in healthcare access and variations in Medicaid expansion, play a critical role in health outcomes [21,22,23]. The Rural Healthy People 2030 Survey emphasizes the importance of enhancing care for individuals with SUDs in rural America, identifying substance use and related topics as priority areas [24]. It specifically identifies substance use prevention, treatment, and recovery services as top priority areas, stressing the urgent need for tailored interventions in these underserved communities. This underscores the necessity of understanding the interplay between substance use, pandemic-related challenges, geographic factors, and demographics to address these priorities effectively.

Prior studies have found an increase in drug and alcohol-related mortality during the pandemic [9,10], with various geographic and demographic factors influencing these trends [9,10,11]. Further, healthcare visits have been proposed as a missed touchpoint in preventing deaths due to drugs and alcohol [25]. Therefore, we hypothesize that healthcare visits where people receive an SUD diagnosis will decrease during the pandemic compared to the year prior to the pandemic. Additionally, the scope and objectives of this study are to investigate healthcare visits for SUDs in the Great Plains region, with a specific focus on alcohol use disorder (AUD), stimulant use disorder (StUD), and opioid use disorder (OUD) during a time period before and during the COVID-19 pandemic. This study aims to assess changes in diagnoses and remission rates during the pandemic, examining variations across demographic groups, including sex, race, ethnicity, rurality, and Medicaid expansion status. While some studies have focused on mortality as an outcome, remission is also a noteworthy outcome to investigate. Remission is determined by the provider’s clinical judgment, but generally indicates a period during which the individual is no longer meeting the diagnostic criteria for the disorder. This can include sustained abstinence from the substance, reduced use, and improvement in related physical and psychological symptoms. Remission does not necessarily mean a complete cure, as there is still a risk of relapse, but it signifies a period of recovery and stability. By exploring these factors, this study seeks to provide a comprehensive understanding of how the pandemic has affected SUD-related healthcare access and outcomes in this predominantly rural area. The ultimate objective is to offer insights into the interplay between substance use, social determinants of health, and geographic challenges, thereby informing targeted programs and policy interventions to better serve underserved populations in the Great Plains. Unlike previous studies, we closely examine how these impacts vary across different demographic groups, including gender, racial and ethnic minorities, rural versus urban residents, and those in Medicaid expansion versus non-expansion states. By focusing on these unique factors, our research fills a critical gap in the existing literature, providing a detailed understanding of how these variables influence SUD treatment access and outcomes during a global health crisis. This insight is crucial for developing targeted, region-specific strategies to improve healthcare delivery and support for these vulnerable populations, especially those residing in rural areas.

## 2. Materials and Methods

### 2.1. Ethics

This study received approval from the Institutional Review Board (IRB) at the University of South Dakota (Vermillion, South Dakota, USA; IRB-21-45; Approved 10 March 2021). The requirement to obtain informed consent was waived, as the study involved data collected for non-research purposes under a category five waiver.

### 2.2. Participants and Records

Sanford Health, the largest rural health provider in the United States, supplied 109,671 patient records of 33,227 individuals diagnosed with alcohol use disorder (AUD), opioid use disorder (OUD), or stimulant use disorder (StUD), indicated by ICD-10 codes F10, F11, and/or F15. The F codification in ICD-10 specifically categorizes different substance use disorders: F10 represents AUD, encompassing various conditions related to problematic alcohol use; F11 denotes OUD, which includes issues related to the misuse of opioids; and F15 identifies StUD, covering disorders associated with the misuse of stimulant substances. The exclusion criteria included being under 18 years old, not receiving care at a Sanford facility, or lacking an ICD-10 diagnosis of F10, F11, and/or F15. Notably, patients could receive multiple SUD diagnoses per visit. We also analyzed remission rates, focusing on ICD-10 codes F10.11, F10.21, F10.91, F11.11, F11.21, F11.91, F15.11, F15.21, and F15.91. The inclusion criteria required a confirmed SUD diagnosis and treatment at a midwestern Sanford Health facility. The participants were from North Dakota, South Dakota, Nebraska, Iowa, Minnesota, Wisconsin, and Illinois. The collected demographic data included age, sex, race, ethnicity, zip code, and state. Records missing data on sex, state, or zip code were excluded, resulting in the exclusion of fifty-two records, as incomplete records could have introduced biases or errors into the analysis, potentially skewing the results and misrepresenting the distribution of SUD diagnoses and other variables. A threshold of 1.5% was set to ensure the privacy and confidentiality of patients. Including race categories with very few individuals could potentially lead to the identification of those patients, especially in a study involving sensitive health information, like SUD diagnoses. By categorizing race groups with less than 1.5% representation as “Other,” we aggregated these smaller groups into a broader category, thus protecting individual identities and adhering to ethical standards in handling patient data. This study focused on adult patients (18 years or older) diagnosed between 1 March 2019 and 31 March 2021. Medicaid expansion status was determined based on the date of the visit. The data were anonymized using the Universal Unique Identifier (UUID). This method generates a unique alphanumeric code for each patient record and ensures that personal identifiers are removed to maintain patient confidentiality.

### 2.3. RUCA Mapping

Zip codes were utilized to assign rural-urban commuting area (RUCA) codes, which define rurality based on population density, urbanization, and commuting patterns [26]. RUCA codes comprehensively classify rural and urban areas, which are critical for understanding healthcare access in different geographic contexts. RUCA codes range from 1 to 10 and were condensed into Metropolitan (RUCA: 1–3) and Non-Metropolitan (RUCA: 4–10) categories to simplify the analysis and enhance the comparability of healthcare access disparities between urban and rural areas. Further, this ensured patient privacy, as visits by patients in some RUCA categories were less than 20 for some of the analyses.

### 2.4. Data Analysis

Logistic regression analyses were performed using SAS Studio to assess the odds of patients being diagnosed with an SUD. This was chosen since the mode number of visits was one per patient. Variables included in the models were sex, race, ethnicity, age, place of residence (Metropolitan vs. Non-Metropolitan), Medicaid expansion status at the time of visit, and pandemic status at the time of visit. The pre-pandemic period was defined as 1 March 2019, to 13 March 2020, and the pandemic period as 14 March 2020, onwards. To analyze changes in remission rates during the pandemic, separate datasets for OUD, AUD, and StUD were created, with remission status (ICD-10 codes F1X.11, F1X.21, F1X.91) as the dependent variable. The same predictor variables were included in these models, as described above. See Appendix A for greater details. Statistical significance was set at *p* = 0.05.

## 3. Results

### 3.1. Visit Characteristics

The demographic distribution of patients closely mirrored the communities served by the healthcare provider. Most visits by patients were people who identified as White (Table 1), followed by Native American, unknown, Black, and Other. A significant majority of visits were non-Hispanic, compared to Hispanic or unknown ethnicity. Visits by female patients comprised 45.84% of the total, while male patients made up 54.16%. Most visits occurred in states where Medicaid had been expanded. A slightly higher proportion of visits were from patients residing in non-metro areas and during the pandemic. The mean age of patients was 46.03 (standard deviation = 15.40). Regarding SUDs, 64.26% of visits were associated with an AUD diagnosis, 26.36% with an OUD diagnosis, and 14.85% with a StUD diagnosis. Additionally, remission diagnoses were noted in 13.56% of AUD cases, 5.78% of OUD cases, and 3.65% of StUD cases. It is important to note that substance use diagnoses were not mutually exclusive. The final number of visits included in the study was 109,619. The majority of patients had only 1 visit (48.09%), with a mean number of visits per patient of 3.86 visits (standard deviation = 11.02; mode = 1; range 1 to 563 visits per patient).

### 3.2. Alcohol Use Disorder

The logistic regression analysis indicated that race significantly predicted an AUD diagnosis. Specifically, patients identified as Native American or Other had lower odds of receiving an AUD diagnosis than White patients (Table 2). In contrast, patients of unknown race had a significantly higher odds ratio compared to White patients, while Black patients did not significantly differ from White patients. Hispanic patients were less likely to receive an AUD diagnosis compared to non-Hispanic patients, while patients of unknown ethnicity had similar odds to non-Hispanic patients. Females were less likely than males to receive an AUD diagnosis. Patients in states with Medicaid expansion were less likely to receive an AUD diagnosis, whereas those residing in metro areas were more likely to receive an AUD diagnosis compared to non-metro patients. The likelihood of receiving an AUD diagnosis was slightly higher during the pandemic compared to the period before it. The odds of receiving an AUD diagnosis also increased with age. The area under the ROC curve for this model was 0.69.

### 3.3. Opioid Use Disorder

The logistic regression analysis for OUD diagnoses (Table 2) revealed significant effects of race, ethnicity, sex, Medicaid expansion, metro residency, pandemic period, and age. Native American and Other race patients had higher odds of receiving an OUD diagnosis compared to White patients. Black patients and those of unknown race had lower odds of receiving an OUD diagnosis compared to White patients. Hispanic and unknown ethnicity patients had higher odds of receiving an OUD diagnosis compared to non-Hispanic patients. Females were 2.7 times more likely than males to receive an OUD diagnosis. OUD diagnoses were less common in states without Medicaid expansion and less likely among metro residents compared to non-metro patients. There was a slight decrease in OUD diagnoses during the pandemic. The probability of receiving an OUD diagnosis increased with age. The area under the ROC curve for this model was 0.71.

### 3.4. Stimulant Use Disorder

The logistic regression analysis for StUD diagnoses (Table 2) identified significant effects of race, ethnicity, sex, Medicaid expansion, pandemic period, and age, but no significant effect of metro residency. Black and unknown-race patients had higher odds of receiving a StUD diagnosis than White patients. Native American and Other race patients had lower odds compared to White patients. Hispanic and unknown ethnicity patients had lower odds compared to non-Hispanic patients. Females had higher odds of receiving a StUD diagnosis than males. Patients in states without Medicaid expansion were less likely to receive a StUD diagnosis. The odds of receiving a StUD diagnosis increased during the pandemic but decreased with age. The area under the ROC curve for this model was 0.69.

### 3.5. Alcohol Use Disorder in Remission

Logistic regression analyses were also conducted to assess factors influencing the odds of receiving a remission diagnosis among patients with identified SUDs (Table 3). For AUD in remission, significant factors included race, ethnicity, sex, Medicaid expansion, metro residency, pandemic period, and age. White patients were more likely to receive an AUD remission diagnosis compared to other races. Non-Hispanic patients were more likely to receive a remission diagnosis than Hispanic and unknown ethnicity patients. Females had higher odds of receiving a remission diagnosis than males. Patients in non-Medicaid expansion states and metro areas had lower odds of receiving a remission diagnosis. The odds of remission were higher before the pandemic. Age increases the likelihood of receiving an AUD remission diagnosis. The area under the ROC for this model was 0.66.

### 3.6. Opioid Use Disorder in Remission

For patients with identified OUD in remission, significant factors included race, ethnicity, sex, Medicaid expansion, metro residency, pandemic period, and age. Native American patients had higher odds of receiving an OUD remission diagnosis than White patients. Other race categories had lower odds than White patients. Hispanic and unknown ethnicity patients were more likely to receive a remission diagnosis than non-Hispanic patients. Females were more likely to receive an OUD remission diagnosis than males. Residing in a Medicaid expansion state increased the odds of receiving a remission diagnosis, while metro residency decreased these odds. The odds of remission were higher pre-pandemic, and older age decreased the likelihood of receiving an OUD remission diagnosis. The area under the ROC was 0.78 for this model.

### 3.7. Stimulant Use Disorder in Remission

In patients with StUD in remission, race, ethnicity, sex, Medicaid expansion, metro residency, and pandemic period were significant factors in receiving a remission diagnosis, while age was not. Black and Native American patients had lower odds of receiving a StUD remission diagnosis compared to White patients. Non-Hispanic patients were more likely to receive a remission diagnosis compared to Hispanic and unknown ethnicity patients. Females had higher odds of receiving a StUD remission diagnosis compared to males. Patients from non-Medicaid expansion states were less likely to receive a remission diagnosis. Metro residency decreased the odds of receiving a StUD remission diagnosis. Remission diagnoses were less common before the pandemic. The area under the ROC curve was 0.65 for this model.

## 4. Discussion

Healthcare visits remain a critical touchpoint and an opportunity to reduce SUD-related mortality [25]. This study examines changes in healthcare visits for SUDs and remission prior to and during the COVID-19 pandemic in the Great Plains, with a focus on identifying the characteristics of those served. Key findings include greater odds of AUD and StUD diagnoses during the pandemic, while OUD diagnoses lessened. Additionally, individuals living in metropolitan areas were more likely to receive an AUD diagnosis, whereas those in non-metropolitan areas were more likely to be diagnosed with OUD. Factors such as being female, residing in a Medicaid expansion state, and living in non-metropolitan areas were associated with higher remission rates for SUDs. Finally, pre-existing factors, like geographic location and demographic characteristics, continued to significantly influence healthcare outcomes for individuals with SUDs.

### 4.1. Alcohol Use Disorder Diagnoses

Alcohol remains a serious healthcare concern and a driver of healthcare related to SUDs in the Great Plains [27]. The current study found that visits of people with AUD increased during the pandemic, were higher in people residing in urban areas, and were more common in males and non-Medicaid-expanded states. These align with alcohol-related mortality data, suggesting that alcohol-related liver mortality was higher in states where Medicaid was not expanded and increased during the pandemic [28]. Similarly, alcohol-related mortality was higher in males compared to females [29]. However, Spencer and colleagues found higher mortality for people residing in rural locations [29]. In contrast to mortality, other studies have also seen increased emergency department visits related to alcohol among metropolitan residents [30]. Moreover, emergency department visits for people diagnosed with AUD were more expensive than those diagnosed with OUD and had a higher fatality rate [30]. Similar findings were seen in Kentucky, but authors noted that access to treatment was also higher in metro locations [31]. We speculate that the difference in the impact of rurality on AUD-related visits compared to AUD-related mortality may be due to rural residents not seeking care or receiving screening for AUD. Prior studies have highlighted the continued struggle for access to care for AUD among rural populations, including barriers to care such as stigma [32]. Finally, the perceived need for treatment may differ among the populations included in the current study. Among South Dakota residents who had a positive screen for alcohol or SUDs, 98.1% did not perceive a need for care [33]. Initiative-taking interactions, such as screenings at wellness visits, as well as greater information about treatments for AUD, may be important, especially among states where AUD-related mortality is high [28].

### 4.2. Opioid Use Disorder Diagnoses

Policy changes may have improved opportunities for the care of patients with OUD. Patients who had visits where an OUD diagnosis was provided were more likely to be female, Native American, or Hispanic and residing in a non-metro or Medicaid-expanded geographical area. This is consistent with our previously published findings that Native American, rural, and female patients were seeking buprenorphine treatment for OUD [34]. Interestingly, visits for OUD were higher prior to the pandemic compared to during the pandemic in the current study. However, buprenorphine prescriptions increased during the pandemic in the same population served by this healthcare system [34]. Findings from these two studies suggest a significant unmet need for the treatment of OUD among rural, female, and/or Native American or Hispanic patients prior to the pandemic. Indeed, limited access to pharmacotherapies for OUD has been noted in the Great Plains [35]. Increased access to care through policy changes and increased participation by practitioners in rural settings contributed to increased buprenorphine prescribing during the pandemic in these populations who faced unmet treatment needs. Thus, we speculate there was a slight reduction in visits of people who meet the diagnostic criteria for OUD during the pandemic in the current study because they had greater access to care for their OUD. The observed decrease in OUD diagnoses during the pandemic could be attributed to several factors. The increased use of telemedicine and the expansion of medication-assisted treatment (MAT) services, such as buprenorphine, may have reduced the need for traditional in-person visits. Additionally, the stigma associated with seeking treatment for OUD, especially in rural areas, may have led to the under-reporting of cases. Further research is needed to explore these dynamics and to identify strategies to improve reporting and treatment access. Visits by people diagnosed with OUD were also higher in Medicaid expansion states. Historically, Medicaid expansion has provided patients with greater SUD treatment access for those who otherwise would not be covered by insurance [36,37,38]. In general, greater access to care, including harm reduction practices, was noted in Medicaid expansion states [38], leading to similar or reduced mortality due to drug overdoses [36,39]. More recently, Medicaid has been expanded in both Nebraska and South Dakota, which may further increase access to care for people with OUD. Although we are hopeful that recent changes will lead to persistent improvements in care for patients with OUD in the Great Plains, sustained efforts to improve care for people with OUD and evaluation of outcomes are warranted.

We also speculate that one possible explanation is the increased availability and accessibility of telehealth services, which may have facilitated better management of OUD symptoms outside traditional clinical settings. Telehealth can provide continuity of care, particularly for maintenance therapies like buprenorphine, which may reduce the need for in-person diagnostic visits. Additionally, the relaxation of regulations around telemedicine and MAT during the pandemic likely played a role in sustaining patient care, as these changes allowed for more flexible prescribing practices and reduced barriers to accessing treatment. Another factor could be the potential under-reporting or misclassification of OUD cases due to changes in healthcare utilization patterns during the pandemic. Patients may have been less likely to seek in-person care due to fear of contracting COVID-19, leading to fewer formal diagnoses despite continued substance use. Investigating the long-term effects of telehealth and regulatory adjustments on OUD treatment outcomes could provide valuable insights into optimizing care delivery in post-pandemic settings. Additionally, exploring the impact of socioeconomic and demographic factors on healthcare-seeking behavior during the pandemic could help identify vulnerable populations who may have experienced disruptions in care. Understanding these nuances is crucial for developing targeted interventions to ensure continued access to OUD treatment and support, particularly in times of public health crises.

### 4.3. Stimulant Use Disorder Diagnoses

Ensuring care for StUD patients is critical because it is becoming more common and associated with more risks [40]. Visits for StUD increased during the pandemic and were more common among people who resided in a Medicaid expansion state. Prior studies suggest that people with this disorder are also more likely to be uninsured or insured by Medicaid [40]. Medicaid expansion may have allowed for greater access to care among people with StUD, resulting in more visits recorded in the current study. Moreover, studies suggest that the demographics using stimulants, primarily methamphetamine, may be changing. Increases in methamphetamine use disorder over time were larger in magnitude among Black individuals [40], which is consistent with the higher odds for this group observed in the current study. Moreover, the use and serious outcomes associated with StUD have been increasing. Consistent with the findings of the present study, StUD, primarily methamphetamine use disorder, and overdose deaths have increased in recent times [40,41]. It is not simply an increase in use statistics that is worrisome, but also how these drugs are being taken. Increases in risky drug-taking behavior, such as injecting, were also noted in people who use methamphetamine [40,42]. These findings highlight the critical need to implement harm reduction for people who use stimulants [41].

### 4.4. Remission Diagnoses

Substance use disorders are viewed as chronic, long-term diseases that require continued care [43,44]. As such, it is essential to understand what factors facilitate continued care. Remission diagnoses for SUDs were also examined as a metric of continued care. For people with SUDs to receive this diagnosis, healthcare providers must follow up with patients on their current health status related to SUDs. Across the SUDs investigated, visits with a remission diagnosis were higher in Medicaid expansion states. Medicaid expansion provides a critical lifeline to healthcare for SUD and other health-related services [36]. Findings suggest that Medicaid expansion helped provide continuing care for those in recovery. Like Medicaid expansion, remission diagnoses were higher for patients residing in non-metro locations. This is a novel finding. Few studies have investigated remission diagnoses and fewer have looked at rurality. Living in a non-metro location was also associated with higher odds of self-reporting being in remission [45]. Studies have noted greater patient care follow-up and care transition scores in non-urban hospitals than in urban hospitals [46]. The authors noted that rural environments may facilitate communication. While the context of care sought differed from the current study, it is conceivable that similar rural environment factors may help support follow-up care of SUD. The higher remission rates observed in rural areas compared to urban settings may be influenced by unique challenges faced by rural populations, including limited healthcare infrastructure and stronger community support networks. These factors, while often seen as barriers, can paradoxically enhance the quality of care through more personalized and continuous interactions between patients and healthcare providers [47]. In rural settings, the scarcity of healthcare facilities often necessitates that providers take on a more holistic role, fostering stronger patient-provider relationships and facilitating better follow-up care [48]. This environment can lead to greater patient engagement and adherence to treatment plans, which are crucial for achieving remission in SUDs. Moreover, the close-knit nature of rural communities may provide a support system that complements medical care, helping patients maintain recovery. Thus, while rural areas face significant healthcare challenges, the dynamics of patient care in these regions may offer advantages in terms of continuity and quality of care, contributing to the observed higher remission rates [49,50]. Finally, females were more likely to receive a remission diagnosis. Prior studies suggest females are more likely to engage in routine healthcare and to have a primary healthcare provider [51,52]. These routine care visits by primary providers may provide opportunities to follow up on patients’ healthcare needs, including the need for further SUD care. Given the importance of continuing care as a critical component of the effective treatment of SUD [43], further research is needed to understand best practices for continuing care of patients with SUDs.

### 4.5. Potential Implications

In addition to examining remission diagnoses, it is crucial to explore the disparities in access to SUD treatment and the factors influencing these differences. Previous studies have highlighted the role of socioeconomic status, insurance coverage, and geographic barriers in shaping healthcare access for SUD patients [49,50,53]. Individuals from lower socioeconomic backgrounds often face financial constraints that limit their ability to seek treatment, even when services are available. Lack of insurance coverage further exacerbates this issue, as uninsured patients are less likely to access both preventive and ongoing care for SUDs. Geographic barriers, such as living in rural or underserved areas, can also impede access to specialized treatment facilities because of transportation, making it challenging for patients to receive timely and appropriate care. The current study’s findings align with this literature, demonstrating that Medicaid expansion and living in non-metro areas were associated with higher remission diagnoses. This underscores the importance of policy interventions in bridging the gap in healthcare access, particularly for underserved populations [54]. Moreover, the observed gender differences in remission rates suggest that women may be more proactive in seeking care, possibly due to higher engagement in routine healthcare practices [55]. This insight supports the need for targeted interventions that address gender-specific barriers and facilitators in SUD treatment. Future research should further investigate these dynamics to inform policies that ensure equitable access to care for all individuals with SUDs, regardless of their demographic or geographic background [56].

The findings of this study have significant policy implications, particularly in addressing the healthcare needs of rural populations and various demographic groups. The increased odds of SUD diagnoses, including AUD and StUD, during the pandemic underscore the necessity for targeted interventions and support mechanisms. In rural areas where healthcare access is often limited, expanding telehealth services and ensuring robust internet infrastructure could enhance the delivery of care. Additionally, the data indicating higher remission diagnoses in Medicaid expansion states suggest that expanding Medicaid coverage could be a critical policy measure to improve healthcare access and continuity of care for individuals with SUDs. Policymakers should consider the benefits of making telemedicine services permanent and integrating them into standard healthcare practices, especially for managing chronic conditions like SUDs. Furthermore, the disparities observed across different racial and ethnic groups highlight the need for culturally competent care and community-specific outreach programs. These should focus on reducing stigma and barriers to accessing treatment, particularly among minority populations who may be underserved. By leveraging these insights, healthcare policies can be tailored to enhance service delivery, reduce health disparities, and ultimately improve health outcomes for individuals with SUDs across diverse settings.

### 4.6. Limitations

While these findings are promising, the study’s limitations should be considered. While the healthcare system is the largest rural health provider, the data are not inclusive of all visits related to SUDs in the Great Plains. The exclusion criteria could introduce biases by potentially under-representing these groups and their specific SUD outcomes. This approach may mask differences in how smaller demographic groups experience and access treatment for SUDs, thereby limiting the study’s generalizability. The Veterans Association, Indian Health Services, and other hospital systems treated patients for SUD during this time. Further, complete medical histories of each patient were not available. We, therefore, cannot distinguish between the impact of the pandemic on the new onset of SUDs and total diagnoses. Future research is necessary to differentiate this. In interpreting the study’s results, it is crucial to distinguish between statistical significance and clinical relevance. Variables with ORs close to one, despite being statistically significant, indicate a minimal practical change in the risk of SUD diagnoses. This distinction is vital to avoid over-interpreting findings that, while statistically notable, do not translate into significant changes in clinical practice or patient outcomes. Therefore, our discussion emphasizes results that have a substantial clinical impact, ensuring that the focus remains on actionable and meaningful insights for healthcare providers and policymakers. Additionally, evidence suggests that not all people who identify as having SUD are diagnosed with the disease [57]. We suspect this is true for the population observed in the current study, especially those with limited access to care. Furthermore, while visits differed, the reason behind these differences is unclear. It is speculated that policies such as Medicaid expansion may impact access to healthcare related to SUD. However, it cannot be ruled out that other factors, such as distance to providers and perceived reduction in stigma, may play a role in states where Medicaid is expanded. The intertwined effects of these elements underscore the complexity of healthcare access and utilization in the context of SUD treatment. Given these considerations, there is a pressing need for follow-up studies that delve deeper into the dynamics behind the observed trends. Such research should aim to disentangle the contributions of policy changes, geographical accessibility, and stigma reduction to better understand their roles in shaping SUD treatment access and utilization. By addressing these gaps, future research can offer more definitive insights, guiding targeted interventions to improve SUD healthcare delivery in rural settings.

## 5. Conclusions

The current study investigated healthcare visits where people received a diagnosis of SUD in the Great Plains. Findings highlight the need for care, especially among people with AUD, and emerging changes for people with StUD. Some metrics of SUDs changed during the pandemic in the Great Plains, such as decreased visits for OUD. Other factors continued to impact healthcare visits for SUDs. Factors related to social determinants of health, such as rurality and residing in a Medicaid-expansion state, also played a vital role. These findings highlight the continued need to investigate methods to increase access to care for SUDs, especially for those facing barriers.

## Figures and Tables

**Table 1:** Demographic and Visit Information.

Characteristic	Percentage (%)	Statistical Values
**Race**		
White	70.83	χ(4) = 192,294.94, *p* < 0.05
Native American	21.39	
Unknown	5.54	
Black	1.72	
Other	0.52	
**Ethnicity**		
Non-Hispanic	91.70	χ(2) = 168,149.96, *p* < 0.05
Hispanic	2.94	
Unknown Ethnicity	5.36	
**Sex**		
Female	45.84	χ(1) = 758.925, *p* < 0.05
Male	54.16	
**Medicaid Expansion State**		
Yes	81.74	χ(1) = 44,184.53, *p* < 0.05
No	18.26	
**Metro/Non-Metro**		
Metro	46.32	χ(1) = 592.778, *p* < 0.05
Non-Metro	53.68	
**Pandemic Period**		
During the pandemic	52.23	χ(1) = 217.69, *p* < 0.05
Prior to the pandemic	47.77	
**Substance Use Disorder Diagnosis**		
AUD	64.26	
OUD	26.36	
StUD	14.85	
**Remission Diagnosis**		
AUD	13.56	
OUD	5.78	
StUD	3.65	
**Number of Visits Per Patients**		
1	48.09	
2–10	45.51	
10+	6.4	

**Table 2:** SUD Models.

Variable		Alcohol Use Disorder (AUD)	Opioid Use Disorder (OUD)	Stimulant Use Disorder (StUD)
		Variable χ² values and Odds Ratio (95% Confidence Limits)	Variable χ² values and Odds Ratio (95% Confidence Limits)	Variable χ² values and Odds Ratio (95% Confidence Limits)
Race		χ²(4) =630.08, *p* < 0.05	χ²(4) = 1469.50, *p* < 0.05	χ²(4) = 102.29, *p* < 0.05
	Black	0.914 (0.824, 1.015)	0.660 (0.574, 0.758) *	1.198 (1.062, 1.352) *
	Native American	0.681 (0.659, 0.703) *	1.794 (1.734, 1.856) *	0.865 (0.829, 0.903) *
	Other	0.513 (0.430, 0.612) *	2.172 (1.815, 2.599) *	0.717 (0.566, 0.907) *
	Unknown	1.480 (1.268, 1.727) *	0.308 (0.255, 0.372) *	1.773 (1.466, 2.144) *
	White (reference)			
Ethnicity		χ²(2) = 389.48, *p* < 0.05	χ²(2) = 580.05, *p* < 0.05	χ²(2) = 28.92, *p* < 0.05
	Hispanic	0.459 (0.425, 0.496) *	2.630 (2.431, 2.845) *	0.757 (0.683, 0.840) *
	Unknown	0.928 (0.794, 1.084)	1.274 (1.059, 1.533) *	0.812 (0.668, 0.987) *
	Non-Hispanic (reference)			
Sex		χ²(1) = 5008.49, *p* < 0.05	χ²(1) = 4404.82, *p* < 0.05	χ²(1) = 115.30, *p* < 0.05
	Female	0.386 (0.376, 0.397) *	2.675 (2.599, 2.754) *	1.211 (1.169, 1.253) *
	Male (reference)			
Medicaid Expansion		χ²(1) = 383.57, *p* < 0.05	χ²(1) = 301.22, *p* < 0.05	χ²(1) = 46.22, *p* < 0.05
	No	1.436 (1.385, 1.489) *	0.695 (0.667, 0.724) *	0.849 (0.810, 0.890) *
	Yes (reference)			
Metro		χ²(1) = 2380.43, *p* < 0.05	χ²(1) = 2671.93, *p* < 0.05	χ²(1) = 0.21, ns
	Metro	1.969 (1.916, 2.023) *	0.449 (0.435, 0.463) *	0.992 (0.957, 1.028)
	Non-Metro (reference)			
Pandemic		χ²(1) = 12.97, *p* < 0.05	χ²(1) = 32.07, *p* < 0.05	χ²(1) = 6.67, *p* < 0.05
	Pandemic	1.049 (1.022, 1.077) *	0.921 (0.895, 0.947) *	1.046 (1.011, 1.083) *
	Pre-Pandemic (reference)			
Age (years)		χ²(1) = 558.12, *p* < 0.05	χ²(1) = 236.28, *p* < 0.05	χ² (1) = 5035.12, *p* < 0.05
		1.011 (1.010, 1.012) *	1.008 (1.007, 1.008) *	0.952 (0.951, 0.954) *

* *p* < 0.05.

**Table 3:** Remission Models.

Variable		Alcohol Use Disorder (AUD) in Remission	Opioid Use Disorder (OUD) in Remission	Stimulant Use Disorder (StUD) in Remission
		Variable χ² values and Odds Ratio (95% Confidence Limits)	Variable χ² values and Odds Ratio (95% Confidence Limits)	Variable χ² values and Odds Ratio (95% Confidence Limits)
Race		χ²(4) = 1151.52, *p* < 0.05	χ²(4) = 346.89, *p* < 0.05	χ²(4) = 416.40, *p* < 0.05
	Black	0.663 (0.570, 0.772) *	0.254 (0.132, 0.488) *	0.467 (0.348, 0.627) *
	Native American	0.342 (0.321, 0.364) *	1.683 (1.570, 1.803) *	0.355 (0.321, 0.393) *
	Other	0.435 (0.295, 0.642) *	0.420 (0.292, 0.604) *	1.014 (0.633, 1.625)
	Unknown	0.449 (0.339, 0.594) *	0.081 (0.049, 0.134) *	0.717 (0.489, 1.050)
	White (reference)			
Ethnicity		χ²(2) = 52.27, *p* < 0.05	χ²(2) = 382.92, *p* < 0.05	χ²(2) = 38.18, *p* < 0.05
	Hispanic	0.666 (0.569, 0.780) *	2.647 (2.355, 2.976) *	0.717 (0.563, 0.914) *
	Unknown	0.421 (0.315, 0.563) *	11.708 (7.589, 18.064) *	0.283 (0.187, 0.428) *
	Non-Hispanic (reference)			
Sex		χ²(1) = 751.56, *p* < 0.05	χ²(1) = 165.39, *p* < 0.05	χ²(1) = 70.69, *p* < 0.05
	Female	1.691 (1.629, 1.756) *	1.555 (1.454, 1.663) *	1.378 (1.278, 1.484) *
	Male (reference)			
Medicaid Expansion		χ²(1) = 177.08, *p* < 0.05	χ²(1) = 51.37, *p* < 0.05	χ²(1) = 31.44, *p* < 0.05
	No	0.714 (0.679, 0.750) *	0.606 (0.529, 0.695) *	0.728 (0.652, 0.814) *
	Yes (reference)			
Metro		χ²(1) = 195.77, *p* < 0.05	χ²(1) = 226.86, *p* < 0.05	χ²(1) = 129.69, *p* < 0.05
	Metro	0.765 (0.737, 0.794) *	0.505 (0.462, 0.552) *	0.642 (0.595, 0.693) *
	Non-Metro (reference)			
Pandemic		χ²(1) = 75.47, *p* < 0.05	χ²(1) = 141.11, *p* < 0.05	χ²(1) = 4.03, *p* < 0.05
	Pandemic	0.848 (0.817, 0.880) *	0.689 (0.648, 0.733) *	1.078 (1.002, 1.161) *
	Pre-Pandemic (reference)			
Age (years)		χ²(1) = 86.80, *p* < 0.05	χ²(1) = 1190.48, *p* < 0.05	χ²(1) = 0.98, ns
		1.006 (1.005, 1.007) *	0.958 (0.955, 0.960) *	1.002 (0.998, 1.005)

* *p* < 0.05.

## Data Availability

The data supporting this study’s findings are available from Sanford Health, but restrictions apply to their availability. These data were used under license for the current study and are not publicly available. Data are, however, available from the authors upon reasonable request and with permission of Sanford Health and the Institutional Review Board at the University of South Dakota. De-identified data can be shared following the completion of a data use agreement that ensures compliance with privacy regulations and ethical standards.

## Appendix A

### Appendix A.1. Supplemental Materials

#### Appendix A.1.1. Model Variables

Variables included in Table 2 were receiving the diagnosis of interest (for example, F10.XXX for Alcohol Use Disorder (AUD) or not receiving this diagnosis at the visit. Similarly, for the Opioid Use Disorder (OUD), the primary outcome of interest was receiving an F11.XXX diagnosis at the visit or not receiving this diagnosis. Finally, for the stimulant use disorder model, the primary outcome was receiving a Stimulant Use Disorder (StUD) diagnosis (F15.XXX) or not receiving this diagnosis at the time of the visit. For the remission models, all patients who received an AUD diagnosis (F10.XXX) were included in the model. Patients who received an AUD in remission diagnosis at that visit (F10.11, F10.21, F10.91) were compared to all other AUD diagnoses. Similarly, patients who received an OUD in remission diagnosis at that visit (F11.11, F11.21, F11.91) were compared to all other visits where an OUD diagnosis was provided. Finally, patients who received a StUD in remission diagnosis at that visit (F15.11, F15.21, F15.91) were compared to all other visits where a StUD diagnosis was provided. Please note, given a full case history was not available for each visit, it is unknown if the diagnoses were new.

Predictor variables included sex, race, ethnicity, age at the time of visit, Medicaid expansion status at the time of visit, pandemic status, and metro/non-metro. Sex was defined as the sex at birth resulting in the responses of male, female, other, and unknown. Visits where other or unknown responses were provided were excluded from the analysis, resulting in 0.08% of visits being excluded. Race was defined as the patient’s first race. Responses included African American, American Indian, Asian, Caucasian/White, Declined, Hispanic/Latino, Native Hawaiian, Pacific Islander, and unavailable/unknown/missing. Given the few responses in the Asian, Hispanic/Latino, Native Hawaiian, and Pacific Islander categories, these were collapsed into the “Other” category, resulting in the Race categories included in the model of White, Native American, Black, Other, and unknown. Patient ethnicity responses included Hispanic or Latino, not Hispanic or Latino, declined, and unspecified/unavailable/unknown/missing. Declined and unspecified/unavailable/unknown/missing were combined into the unknown ethnicity category, resulting in Hispanic or Latino, not Hispanic or Latino, and unknown ethnicity being included in the model. Age was defined as the patient’s age at the time of visit derived from the date of birth provided. Medicaid expansion status was derived from the state of residency reported by the patient and the status of Medicaid expansion in that state at the time of the visit. Pandemic status was derived from the date of the visit. The pre-pandemic period was defined as 1 March 2019, to 13 March 2020, and the pandemic period as 14 March 2020, onwards. This was based on the Trump Administration declaring SARS-CoV-2 a nationwide emergency on 13 March 2020. Zip codes for the patient’s self-reported residence were utilized to assign rural-urban commuting area (RUCA) codes, which define rurality based on population density, urbanization, and commuting patterns. Metropolitan (RUCA: 1–3) and non-metropolitan (RUCA: 4–10) categories were separated to simplify the analysis and enhance the comparability of healthcare access disparities between urban and rural areas. This aligns with the U.S. Department of Agriculture’s (USDA)’s Economic Research Service (ERS) definition of metropolitan/non-metropolitan and helps ensure patient privacy for those residing in RUCA codes with few visits and ensures greater than 20 visits per geographic category.

#### Appendix A.1.2. Rationale for Models

We examined SUD diagnoses and remission diagnoses during a period that spanned one year prior to the pandemic and the first year of the pandemic. It assessed these metrics in the Great Plains, an area of the country with pre-existing challenges in accessing treatment for SUD. The inclusion of variables such as sex, race, ethnicity, age, place of residence, Medicaid expansion status, and pandemic status in the study was carefully considered to comprehensively analyze their impact on substance use disorder (SUD) outcomes. Sex and age were included due to their correlation with different patterns and severities of SUDs; certain age groups or genders may be more prone to specific substances. Race and ethnicity were crucial to identifying potential disparities in diagnosis and treatment, as these factors can highlight systemic biases and social determinants affecting healthcare access. The differentiation between metropolitan and non-metropolitan areas was vital to assess geographical barriers, with rural areas often facing limited healthcare facilities and resources, impacting the quality and availability of treatment. Medicaid expansion status was included to evaluate the effects of policy changes regarding healthcare access, as expanded Medicaid coverage typically leads to broader availability of services. The pandemic status was an essential temporal factor, allowing the study to explore how the COVID-19 pandemic influenced healthcare utilization and SUD management.

In our study, logistic regression was utilized to determine the odds of being diagnosed with SUDs based on a range of predictors, including sex, race, ethnicity, age, place of residence, Medicaid expansion status, and pandemic status. This statistical method is particularly suited for binary outcomes, such as whether a patient is diagnosed with an SUD. By quantifying the influence of each predictor, logistic regression allows us to identify which factors significantly affect the likelihood of an SUD diagnosis. This approach is crucial for highlighting disparities in healthcare access and outcomes among different demographic groups. The findings from the logistic regression analysis can guide targeted interventions and policy changes aimed at reducing these disparities and improving care for individuals with SUDs. The inclusion of a variety of predictors ensures a comprehensive understanding of the factors influencing SUD diagnoses, making the analysis robust and relevant for informing healthcare strategies.
